# Essential elements of radical pair magnetosensitivity in *Drosophila*

**DOI:** 10.1038/s41586-023-05735-z

**Published:** 2023-02-22

**Authors:** Adam A. Bradlaugh, Giorgio Fedele, Anna L. Munro, Celia Napier Hansen, John M. Hares, Sanjai Patel, Charalambos P. Kyriacou, Alex R. Jones, Ezio Rosato, Richard A. Baines

**Affiliations:** 1 Division of Neuroscience, School of Biological Sciences, Faculty of Biology, Medicine and Health, University of Manchester, Manchester Academic Health Science Centre, Manchester, UK; 2 Department of Genetics and Genome Biology, University of Leicester, Leicester, UK; 3 Department of Biology, Ecology and Earth Sciences (DiBEST), University of Calabria, Rende, Italy; 4 Pelican Healthcare, Cardiff, UK; 5 Manchester Fly Facility, Faculty of Biology, Medicine and Health, University of Manchester, Manchester, UK; 6 Biometrology, Chemical and Biological Sciences Department, National Physical Laboratory, Teddington, UK

**Keywords:** Molecular neuroscience, Sensory processing

## Abstract

Many animals use Earth’s magnetic field (also known as the geomagnetic field) for navigation^1^. The favoured mechanism for magnetosensitivity involves a blue-light-activated electron-transfer reaction between flavin adenine dinucleotide (FAD) and a chain of tryptophan residues within the photoreceptor protein CRYPTOCHROME (CRY). The spin-state of the resultant radical pair, and therefore the concentration of CRY in its active state, is influenced by the geomagnetic field^2^. However, the canonical CRY-centric radical-pair mechanism does not explain many physiological and behavioural observations^2–8^. Here, using electrophysiology and behavioural analyses, we assay magnetic-field responses at the single-neuron and organismal levels. We show that the 52 C-terminal amino acid residues of *Drosophila melanogaster* CRY, lacking the canonical FAD-binding domain and tryptophan chain, are sufficient to facilitate magnetoreception. We also show that increasing intracellular FAD potentiates both blue-light-induced and magnetic-field-dependent effects on the activity mediated by the C terminus. High levels of FAD alone are sufficient to cause blue-light neuronal sensitivity and, notably, the potentiation of this response in the co-presence of a magnetic field. These results reveal the essential components of a primary magnetoreceptor in flies, providing strong evidence that non-canonical (that is, non-CRY-dependent) radical pairs can elicit magnetic-field responses in cells.

## Abstract (web summary)

Non-CRYPTOCHROME-dependent radical pairs can elicit responses to magnetic fields in neurons in *Drosophila melanogaster*.

## Main

The ability of species to navigate considerable distances has long intrigued the biological community^1^. One of several environmental cues to support these migrations is the geomagnetic field (geoMF). Moreover, several other behaviours respond reliably to magnetic fields (MFs), at least under laboratory conditions, showing that the ability to sense and react to MFs is not limited to migrating animals^9^. However, the identity of the primary magnetoreceptors, the mechanisms that underlies their reported light dependence and how the magnetic signal is transduced remain unclear^10,11^. A favoured model posits a light-induced electron-transfer reaction whereby radical pairs (RPs) are formed, the spin-states of which are sensitive to MFs as small as the geoMF (around 50 μT)^2^. This so-called RP mechanism (RPM) canonically requires the flavoprotein CRY, which is best known for its role as a circadian blue light (BL) photoreceptor in flies and as a light-insensitive transcriptional regulator in the circadian clock of mammals^2,10^.

Absorption of BL by CRY-bound FAD initiates an electron-transfer cascade along a conserved chain of tryptophan (Trp) residues^2,12–14^. In *Drosophila* this forms a spin-correlated RP comprising the photoreduced FAD (FADº^−^) and the terminal oxidized Trp (TrpHº^+^)^15^. The spin-state of the RP is initially polarized as a singlet (S, anti-parallel spins), which then rapidly oscillates between S and the triplet spin states (T, parallel spins). Transiently (that is, before the system relaxes to equilibrium), this interconversion can be sensitive to MFs, which in turn can lead to downstream modifications in the biological activity of *Drosophila melanogaster* CRY (*Dm*CRY) through conformational change^2^. In its activated state, the *Dm*CRY C-terminal tail of around 20 residues (CTT) becomes exposed, enabling interactions with signalling partners, including PDZ-domain-containing proteins^16–22^.

Although there is ample evidence consistent with CRY being both necessary and sufficient for light-dependent magnetosensitivity, there are a number of studies that support exceptions to this mechanism^2–8^. In one of the most notable, a circadian behavioural assay in *Drosophila* was used to show that *Dm*CRY-dependent light and magnetosensitivity could be rescued in Dm*cry-*null adult flies through expression of the 52 C-terminal (CT) residues of *Dm*CRY fused to GFP (GFP–CT) for stability^3^. Furthermore, *Dm*CRYΔ, resulting from the deletion of the CTT of *Dm*CRY, appeared largely insensitive to an MF, although BL sensitivity was maintained^3,4^.

The *Drosophila* CRY CT lacks both the FAD-binding pocket and the chain of four Trp residues (Trp394, Trp342, Trp397 and Trp420) that are presumed to be necessary for the canonical RPM^2,23–25^. Moreover, mutating these Trp residues, including W420F and W342F, at best attenuates, but does not abolish, the magnetic functionality of *Dm*CRY^3,4,26,27^. These results are inconsistent with the current understanding of the RPM and question the identity of the magnetically sensitive RP in the receptor. Proposed alternatives to a RP between FADº^−^ and TrpHº^+^ include the formation of an RP between FADº^−^/FADHº and O_2_º^−^ or another (unknown, Zº) radical. It is a matter of some contention whether these unconventional RPs contribute to magnetoreception or even represent a primary sensor^11,28–30^.

Here we report the expression of a new transgene encoding *Dm*CRY-CT fused to luciferase (hereafter, Luc–CT), which lacks the canonical FAD-binding pocket and Trp chain and is therefore unable to support light-induced intramolecular electron transfer. Nevertheless, Luc–CT was sufficient to generate changes in BL- and MF-dependent phenotypes in a whole-organism circadian behavioural assay and in the electrophysiological activity of a model neuron, the larval aCC motorneuron. We show that the MF responsiveness of Luc–CT is potentiated by increasing the intracellular concentration of free FAD, to the point at which high levels of this flavin alone can, in the absence of Luc–CT, support an MF response. Finally, we confirm by mutational analysis that the integrity of the CTT of *Dm*CRY correlates with its ability to facilitate sensitivity to an MF. Overall, our results suggest that ‘sensing’ and ‘transducing’ MFs are separate properties that do not need to be carried out by the same molecule.

## *Dm*CRY-CT supports magnetosensitivity

**Fig. 1:**
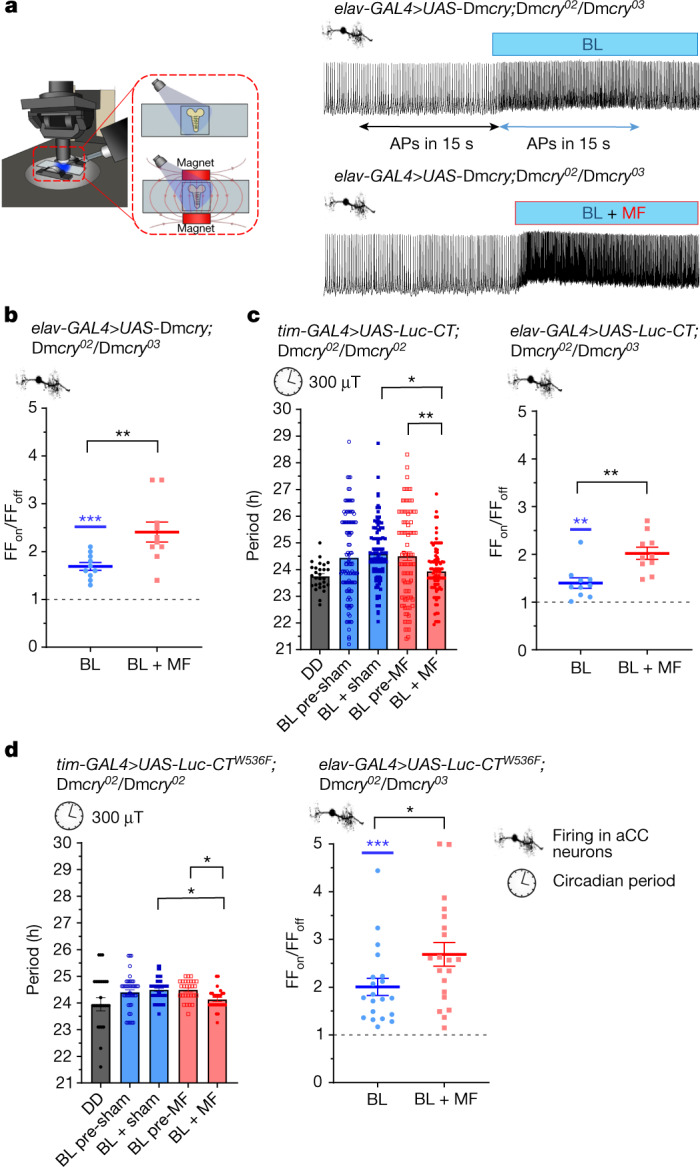
Luc–CT is sufficient to support magnetosensitivity. **a**, The electrophysiology set-up (permanent magnets are shown in red) (left). Top right, BL-exposure of aCC neurons expressing Dm*cry* increases action potential firing. Bottom right, the co-presence of MF (100 mT) potentiates the effect. The traces are from different preparations. APs, action potentials. **b**, The relative firing frequency (FF) of aCC neurons expressing Dm*cry*. BL increases firing 1.69-fold (*t*_9_ = 7.72, *P* ≤ 0.0001, *n* = 10, FF_on_/FF_off_) compared with in the dark (dashed line). External MF (BL + MF, 100 mT) potentiates the effect to 2.41-fold (BL versus BL + MF, *t*_18_ = 3.2, *P* = 0.005, *n* = 10; Extended Data Fig. 1a). **c**, *tim-GAL4>UAS-Luc-CT;*Dm*cry*^*02*^*/*Dm*cry*^*02*^ shows period shortening under MF (left) (sham/MF × before/after exposure interaction (*F*_1,377_ = 7.6, *P* = 0.006, three-way ANOVA). *n* = 28 (DD), *n* = 108 (BL pre-sham), *n* = 93 (BL + sham), *n* = 104 (BL pre-MF), *n* = 90 (BL + MF). MF-exposed flies show a significantly shorter period. Four repeats showed the same period shortening under an MF (Extended Data Table 1). Right, Luc–CT supports BL-induced firing (1.4-fold, *t*_9_ = 4.01, *P* = 0.003, *n* = 10; Extended Data Fig. 1b) potentiated twofold after BL + MF treatment (BL versus BL + MF, *t*_18_ = 3.71, *P* = 0.002, *n* = 10). **d**, Luc–CT(W536F) revealed significant period shortening after exposure to an MF (left) (significant before/after exposure × MF/sham interaction, *F*_1,198_ = 5.1, *P* = 0.025, two-way ANOVA). *n* = 29 (DD), *n* = 47 (BL pre-sham), *n* = 51 (BL + sham), *n* = 52 (BL pre-MF), *n* = 52 (BL + MF). Post hoc tests are shown in Extended Data Table 1. Right, aCC neurons expressing Luc–CT(W536F) show a twofold change in BL-induced firing (*t*_19_ = 6.06, *P* ≤ 0.0001, *n* = 20). The response to BL + MF was variable, but greater than BL alone (2.69-fold, two-way ANOVA, replicates as factor, *F*_1,16_ = 5.09, *P* = 0.03, *n* = 20; Extended Data Fig. 1c). Controls are reported in Extended Data Figs. 3 and 4 and Extended Data Table 3. For FF_on_/FF_off_ data, the blue asterisks represent significance comparing before versus during BL exposure (same cells, paired two-tailed *t*-tests) and the black asterisks represent comparisons of BL versus BL + MF (different cells, unpaired two-tailed *t*-tests). The reported *n* value for each electrophysiological recording is derived from independent cells from biologically independent animals. The reported *n* values for each circadian period derives from biologically independent animals. Data are mean ± s.e.m. *P ≤ 0.05, **P ≤ 0.01, ***P ≤ 0.001.

**Extended Data Fig. 1:**
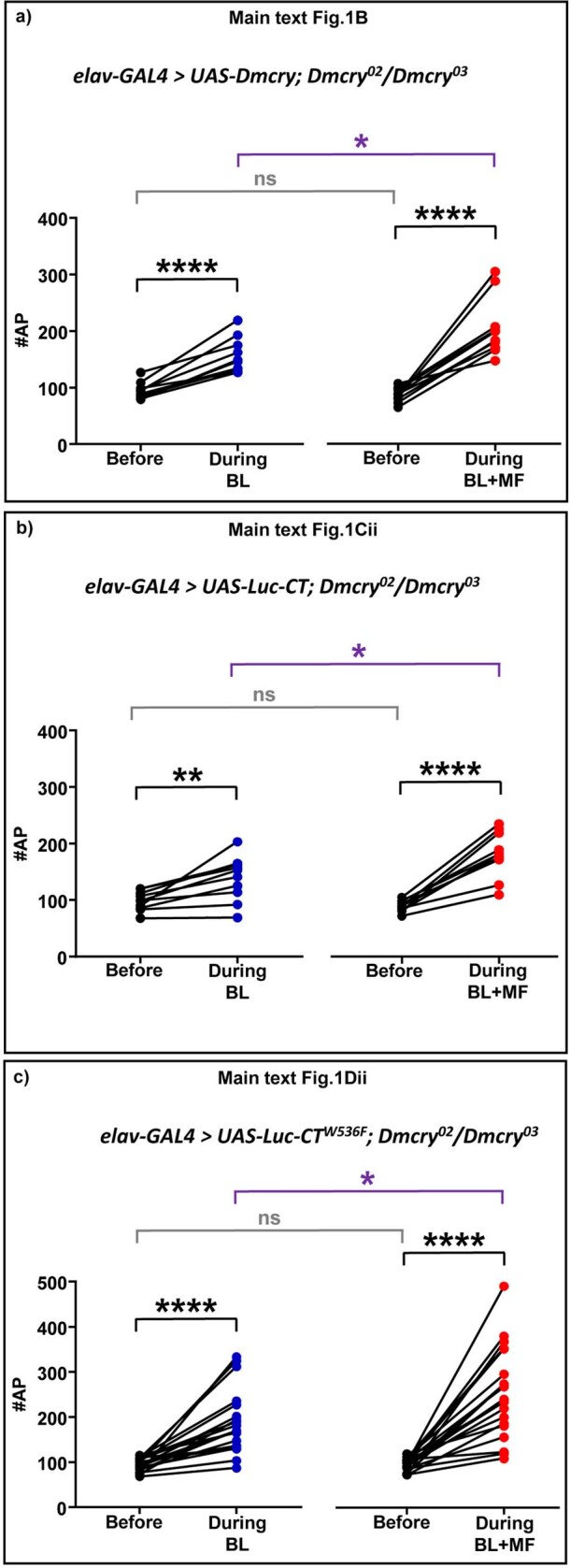
Supporting electrophysiological data for main text Fig. 1. (a). Raw action potential (AP) counts for aCC neurons expressing *Dm*CRY, BL: n = 10, BL+MF: n = 10 (b). Luc-CT, BL: n = 10, BL+MF: n = 10 or (c). Luc-CT^W536F^, BL: n = 20, BL+MF: n = 20, recorded in the 15 s before and 15 s during exposure to BL or BL±MF. These data were used to derive firing-fold (FF) change reported in main text Fig. 1. Paired t-tests – two tailed – were used to compare AP counts before *vs*. during for cells exposed to BL (left hand graph) or to BL±MF exposure (right hand graph). MF-potentiation between the two groups was tested by unpaired t-test – two tailed (different cells). The figure number at the top of each panel represents the main text figure supported. The reported n for each electrophysiological recording is derived from independent cells from biologically independent animals. ns p ≥ 0.06, *p ≤ 0.05, **p ≤ 0.01, ***p ≤ 0.001. See main text Fig. 1 for FF_on_/FF_off_ ratio comparisons.

**Extended Data Fig. 2:**
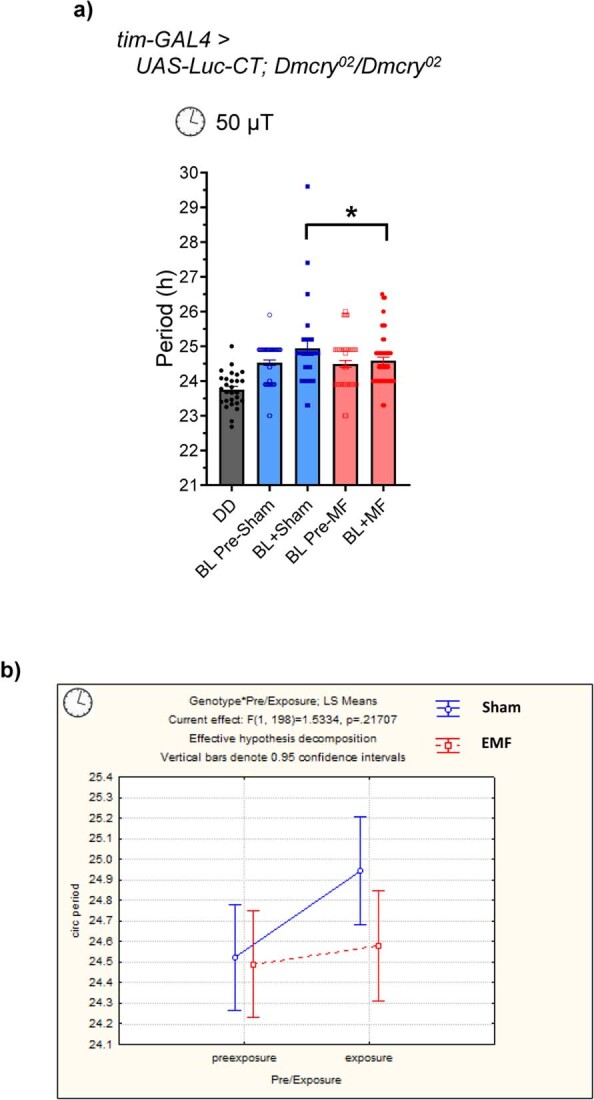
Supporting circadian data for main text Fig. 1. (a). Showing a relative period shortening following expression of Luc-CT under MF (EMF- bars coloured red) compared to the BL+Sham exposure (bars coloured blue). (b). Box and whisker diagram (95% confidence limits) representation of the data shown in the upper panel, omitting DD. Error bars denote ±SEM, ns p ≥ 0.06, *p ≤ 0.05, **p ≤ 0.01, ***p ≤ 0.001.

**Extended Data Fig. 3:**
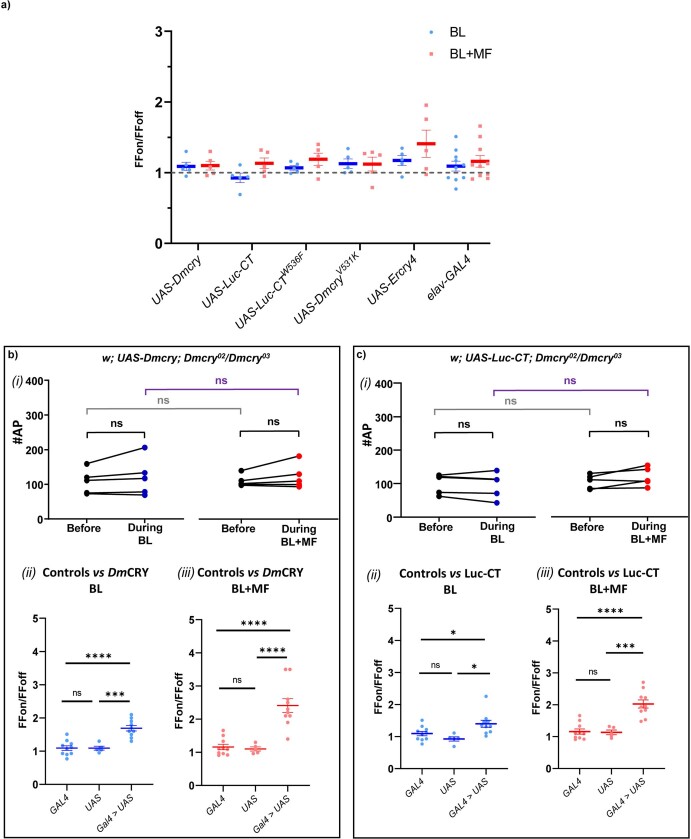
Supporting electrophysiological data (controls). (a). Averaged data for parental (control) *GAL4* and *UAS* genotypes separately in a *Dmcry*-null mutant background. Without the *GAL4* and *UAS* elements combined the *Dmcry-*transgene (under *UAS* control) is not expressed and no significant BL or BL+MF response is seen. A 2-way ANOVA of the controls in both BL or BL+MF showed no significant interaction (F_(4,50)_ = 0.52, p = 0.719), n for all UAS-*Dmcry* transgenic controls in BL and BL+MF = 5. n for *elav-GAL4; ; Dmcry*^*02*^*/Dmcry*^*03*^ driver line BL and BL+MF = 10. (b-c). Raw AP counts *(i)* for each aCC neuron recorded in the 15 s before *vs*. the 15 s during BL or BL±MF exposure for expression of the respective UAS-*Dmcry* transgene stated (paired t-tests – two tailed). As an additional comparison of MF effect, unpaired t-tests – two tailed, were used to determine significant differences between raw AP counts for both ‘before’ exposure conditions (grey line), and for during BL *vs*. BL+MF exposure (purple line). The reported n for each electrophysiological recording is derived from independent cells from biologically independent animals. The reported n for each circadian period derives from biologically independent animals. Error bars denote ±SEM, ns p ≥ 0.06, *p ≤ 0.05, **p ≤ 0.01, ***p ≤ 0.001.

**Extended Data Fig. 4:**
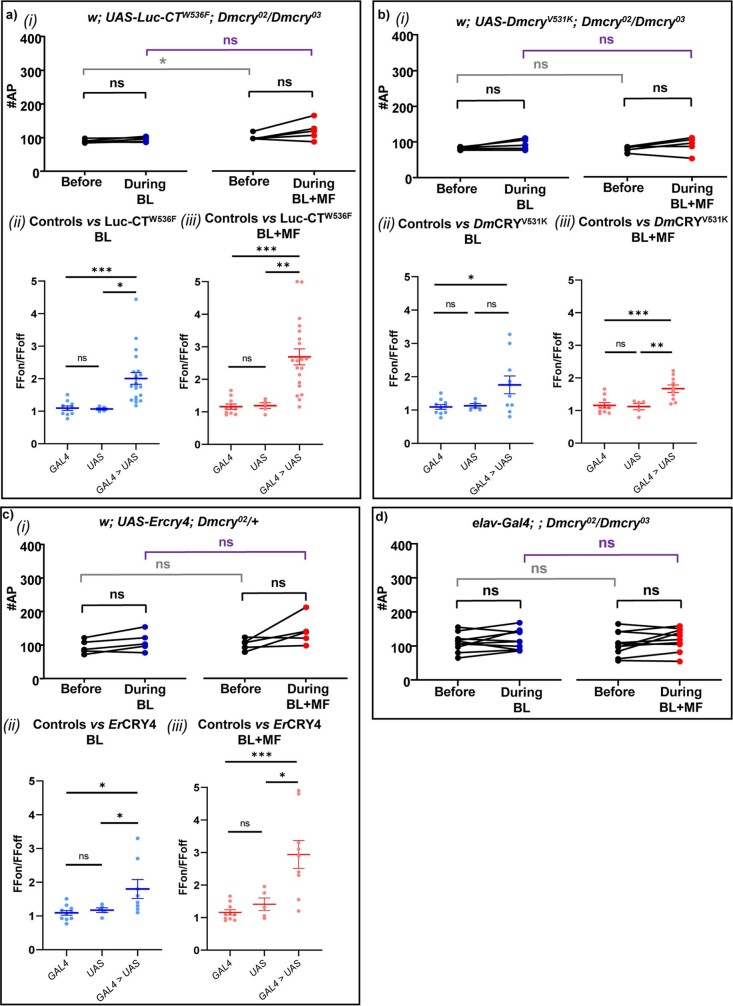
Supporting electrophysiological data (controls). (a–c). Raw AP counts *(i)* for each aCC neuron recorded in the 15 s before *vs*. the 15 s during BL or BL±MF exposure for expression of the respective UAS-*Dmcry* transgene stated (paired t-tests – two tailed). (d). Data for a *Dmcry* null. As an additional comparison of MF effect, unpaired t-tests – two tailed were used to determine significant differences between raw AP counts for both ‘before’ exposure conditions (grey line), and for during BL *vs*. BL+MF exposure (purple line). The reported n for each electrophysiological recording is derived from independent cells from biologically independent animals. The reported n for each circadian period derives from biologically independent animals. Error bars denote ±SEM, ns p ≥ 0.06, *p ≤ 0.05, **p ≤ 0.01, ***p ≤ 0.001.

To validate our electrophysiological assay, we expressed a full-length Dm*cry* transgene in the aCC motorneuron; this supported a BL-induced increase in action potential firing by 1.7-fold and by 2.4-fold, in the co-presence of an MF^4^ (BL + MF, 100 mT static, *P* = 0.005; Fig. 1a,b and Extended Data Fig. 1a). Expression of *Dm*CRY-CT (fused to GFP) in a Dm*cry-null* background supports an MF-induced shortening of the circadian period^3^. To eliminate the possibility that GFP might, like *Dm*CRY, support intramolecular light-induced electron-transfer, we fused *Dm*CRY-CT to luciferase and maintained the flies in the absence of luciferin substrate. BL lengthened the free-running period of *tim-GAL4*>*UAS-Luc-CT;*Dm*cry*^*02*^*/*Dm*cry*^*02*^ flies compared with those in constant darkness (DD) (24.50 h versus 23.75 h, respectively, *P* = 0.019) revealing the BL sensitivity of *Dm*CRY-CT. As with the GFP–CT construct^17^, Luc–CT exposure to an MF (300 µT, 3 Hz) was sufficient to shorten the free-running circadian period in the MF-exposed but not the sham group (0 µT MF; Fig. 1c (left); before exposure/after exposure × sham/MF interaction, *F*_1,377_ = 7.6, *P* = 0.006; Extended Data Table 1a,b). Notably, the Luc–CT fusion supports an MF-mediated period shortening compared with the sham group at 50 µT, which is around the strength of the geoMF (Extended Data Table 2). Expression of Luc–CT in the aCC neuron supported a BL-induced increase in action potential firing (1.4-fold), which was increased further in the co-presence of an MF (100 mT; twofold, *P* = 0.002; Fig. 1c (right) and Extended Data Fig. 1b). In summary, these data collectively show that the Luc–CT alone is sufficient to support magnetosensitivity in both circadian and electrophysiological phenotypes. We predict that it does so through its well-described interaction with the redox-sensitive K^+^ channel β-subunit HYPERKINETIC (HK)^30^. To improve sensitivity when measuring at the single-neuron level, we chose to use a 100 mT moderate field exposure, using permanent magnets, to saturate the Zeeman effect on the radical pair. According to the RPM^31,32^, this is likely to elicit a larger response to MF than exposure to a µT field. We acknowledge that this field strength is greater than the geoMF; however, the observed effects at 100 mT remain consistent with the RPM and our period-shortening assay showed effects at geoMF strength (Extended Data Fig. 2 and Extended Data Table 2a–c). Control data for electrophysiology are reported in Extended Data Figs. 3 and 4 and Extended Data Table 3.

## Trp536 is probably not an RP partner

Although CRY-bound FAD may be dispensable, it is possible that FAD in proximity could interact by forming an RP with the sole (non-canonical) Trp in the CT of *Dm*CRY (Fig. 3a). This alternative mechanism may explain why mutations of single Trp residues that constitute the Trp-tetrad are not entirely detrimental to CRY-dependent magnetoreception^3,4,26,33^. The Trp residue in *Dm*CRY-CT has not been implicated in the canonical RPM. However, it is theoretically capable of generating a RP with free FAD reminiscent of the interaction between Flavin mononucleotide and the surface Trp of lysozyme^34^. Thus, we substituted this residue for a redox-inactive phenylalanine (that is, W536F).

Expression of Luc–CT(W536F) was sufficient to lengthen the circadian period in BL versus DD (24.47 h versus 23.95 h, respectively, *P* = 0.0017) indicating that it supports circadian light responsiveness. A two-way analysis of variance (ANOVA) revealed a significant interaction between pre-exposure/exposure and MF/sham treatment (*F*_1,198_ = 5.1, *P* = 0.025; Fig. 1d (left) and Extended Data Table 1c). Expression of this variant also shortened the free-running circadian period when exposed to an MF (300 µT, 3 Hz) compared with pre-exposure and to sham-exposed flies (*P* = 0.023 and *P* = 0.015, respectively, Fisher least significant difference (LSD) test; the stringent Newman–Keuls test narrowly missed the significance threshold for both comparisons (*P* = 0.063 and *P* = 0.074, respectively); Extended Data Table 1d,e). Expression of Luc–CT(W536F) also supported a strong (twofold) BL response on action potential firing in aCC neurons and again a significant, albeit more variable, potentiation under the BL + MF condition (Fig. 1d (right); 2.69-fold, *P* = 0.03, two-way ANOVA replicates as a factor; Extended Data Fig. 1c). The fact that Luc–CT(W536F) does not abolish an MF response argues against an important role for a hypothetical RP between Trp536 and FAD. Indeed, the weaker MF response may be structural in origin^35^. An arginine (Arg532) in close proximity may form a cation-π interaction with Trp536 to stabilize an alpha-helical conformation^36^ that would be disrupted by the W536F substitution, yet the MF effect is still detectable.

## Free FAD supports magnetosensitivity

**Fig. 2:**
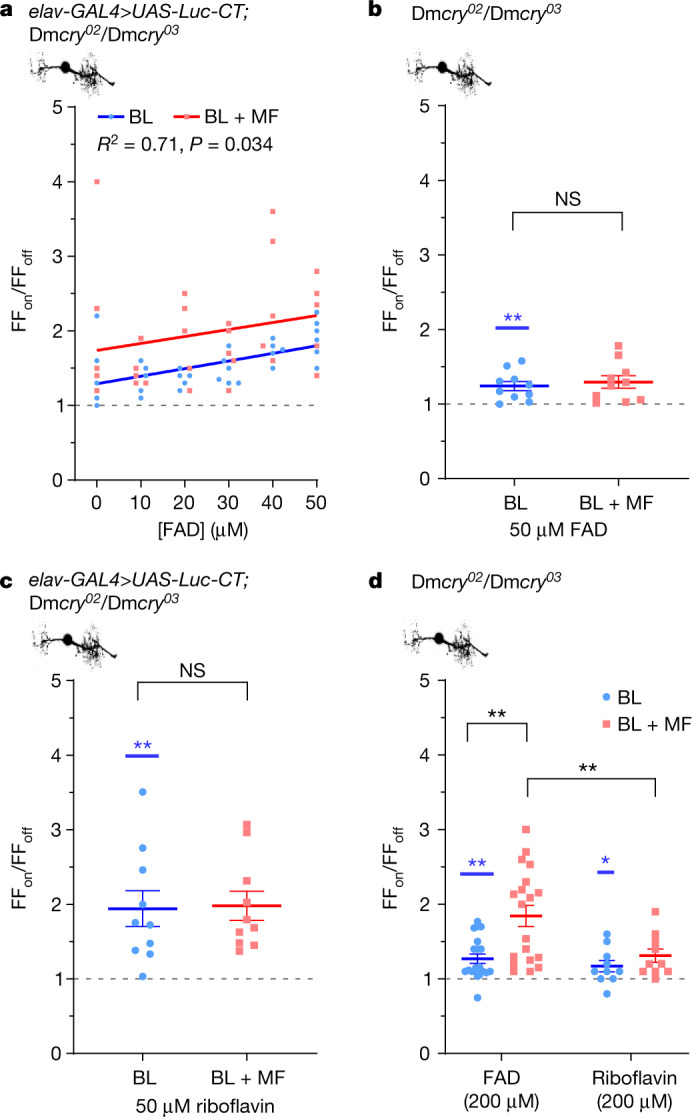
Free FAD potentiates the effect of Luc–CT and, at high concentration, supports magnetosensitivity alone. **a**, Exposing aCC neurons expressing Luc–CT to FAD (through the recording pipette) increases the response to BL (*R*^2^ = 0.71, *F*_1,4_ = 10.1, *P* = 0.03, linear regression). The co-presence of an MF (100 mT) potentiates the response (*F*_1,9_ = 9.06, *P* = 0.015, analysis of covariance (ANCOVA) model). *n* = 5 except for BL 30 µM and 50 µM FAD, for which *n* = 6. **b**, The addition of FAD (50 µM) supports BL sensitivity (Extended Data Fig. 5a), but not magnetosensitivity in the absence of Luc–CT (*t*_18_ = 0.521, *P* = 0.609, *n* = 10). **c**, The addition of riboflavin (50 µM) to aCC neurons expressing Luc–CT, supports the response to BL, but not MF potentiation (*t*_18_ = 0.12, *P* = 0.91). *n* = 10. **d**, Increased FAD (200 µM) in the Dm*cry*^*02*^-null background supports a BL-induced change in firing (1.27-fold, *t*_19_ = 4.29, *P* = 0.0004, *n* = 20; Extended Data Fig. 5c). The co-presence of an MF (*n* = 19) significantly potentiates this effect (1.84-fold, two-way ANOVA, *F*_1,55_ = 3.51, *P* = 0.066, Newman–Keuls post hoc *P* = 0.003). Riboflavin (200 µM) shows a similar BL effect (1.17-fold, *t*_9_ = 2.33, *P* = 0.045, *n* = 10; Extended Data Fig. 5c) but no MF potentiation (1.31-fold, Newman–Keuls post hoc, *P* = 0.67, *n* = 10). Raw data are reported in Extended Data Fig. 5. The blue asterisks represent significance values before versus during BL exposure (same cells, paired two-tailed *t*-tests) and the black asterisks represent comparisons of the BL versus BL + MF condition (different cells, unpaired two-tailed *t*-tests). NS, not significant. The reported *n* value for each electrophysiological recording is derived from independent cells from biologically independent animals. Data are mean ± s.e.m. NS, *P* ≥ 0.06.

**Extended Data Fig. 5:**
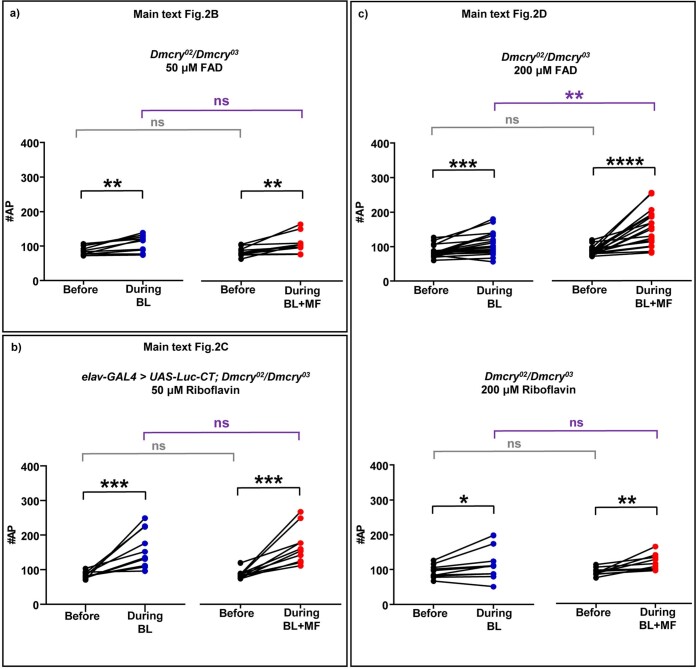
Supporting electrophysiological data for main text Fig. 2. Raw AP counts for averaged data shown for flavin supplementation. (a). *Dmcry*^*02*^*/Dmcry*^*03*^ null cells supplemented with 50 µM FAD show a BL response (n = 10), but no MF potentiation (n = 10) compared to BL alone. (b). Cells expressing Luc-CT supplemented with riboflavin (50 µM) show no MF effect (n = 10) compared to BL (n = 10). (c). Supplemented FAD and riboflavin (200 µM) to *Dmcry*^*02*^*/Dmcry*^*03*^ null cells: FAD supports BL (n = 20) and BL+MF (n = 20) sensitivity, whilst riboflavin only supports BL sensitivity (see main text for BL and BL+MF comparisons). Paired t-tests – two tailed, were used to compare before *vs*. during for cells exposed to BL (left hand graph) or to BL±MF exposure (right hand graph). MF-potentiation between the two groups was tested by unpaired t-tests – two tailed (different cells). The reported n for each electrophysiological recording is derived from independent cells from biologically independent animals. ns p ≥ 0.06, *p ≤ 0.05, **p ≤ 0.01, ***p ≤ 0.001.

The fact that Luc–CT(W536F) is sufficient to support magnetosensitivity implies that a different, non-CRY, RP is involved. In this regard, it is notable that free FAD has the ability to generate a magnetically sensitive RP through intramolecular electron transfer^7,37^. To investigate this, we supplemented additional FAD to aCC neurons through the internal patch saline. Increasing FAD (range, 10 to 50 µM in the patch pipette) potentiates the efficacy of Luc–CT to mediate BL-dependent increases in action potential firing (Fig. 2a; *R*^2^ = 0.71, *P* = 0.034), an effect that is enhanced in the presence of BL + MF (100 mT, *P* = 0.015). Notably, MF potentiation is by a fixed proportion relative to BL at each FAD concentration tested (evidenced by equal gradients of lines of best fit). This is a prediction of the RPM; provided that biological saturation is not limiting, the proportional magnetically induced change should remain constant^31^. In the absence of Luc–CT, FAD (up to 50 µM) induced a weak, but significant, response to BL (*P* = 0.03; Fig. 2b); however, no potentiating effect was observed in the BL + MF condition (Extended Data Fig. 5a).

**Extended Data Fig. 6:**
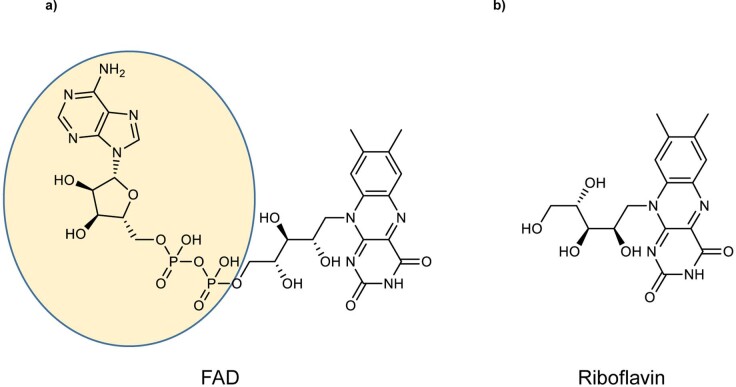
Structures of FAD and Riboflavin. The molecular structure of the two flavin chromophores used. (a). Flavin Adenine Dinucleotide (FAD). Note the adenine diphosphate side chain of FAD (yellow oval), which facilitates the generation of an intramolecular, magnetically-sensitive RP. (b). Riboflavin, which is a metabolic precursor to FAD lacks the diphosphate side chain.

FAD autoreduction occurs after electron transfer from the adenine side chain to the photoexcited isoalloxazine, generating an intramolecular RP^7,38^. To test whether this autoreduction is likely to support magnetosensitivity, we introduced riboflavin to the cell through the internal patch saline. Although riboflavin contains the same isoalloxazine chromophore and can populate photoexcited triplet states^39^, it lacks an adenine diphosphate side chain (Extended Data Fig. 6) and is therefore unable to generate the same intramolecular RP^40^. Riboflavin (50 µM) in the presence of Luc–CT supported a BL effect (~1.94-fold; Fig. 2c), but there was no additional increase under the BL + MF condition (100 mT; *P* = 0.9; Fig. 2c and Extended Data Fig. 5b).

Our results are consistent with an interaction between FAD and Luc–CT, possibly in a complex with other, unknown, molecules, which together may facilitate the transduction of a magnetic field. Furthermore, our data suggest that molecules other than CRY are able to generate magnetically sensitive RPs and produce a biological effect under appropriate conditions. In vitro spectroscopy has shown that BL-photoexcited FAD generates RPs that are responsive to MFs^41^, and it appears probable that FAD is responsible for MF effects that were recently observed on cellular autofluorescence^37^. Thus, FAD (but not riboflavin) at higher concentrations may act as a magnetoreceptor. To test this, we recorded from aCC neurons in the Dm*cry-*null background, which shows no overall BL or MF response (Extended Data Fig. 4d). We observed that high levels of FAD in the internal patch saline (200 µM) were sufficient to support a BL-dependent increase in action potential firing without the need for Luc–CT (1.27-fold; Fig. 2d and Extended Data Fig. 5c). Notably, this effect was potentiated in the presence of an MF (100 mT, 1.84-fold, *P* = 0.003; Fig. 2d). Cells supplemented with riboflavin (200 µM) showed an increase in action potential firing in response to BL (Fig. 2d and Extended Data Fig. 5c) but did not show potentiation of the response in an MF (100 mT, *P* = 0.67). The fact that high levels of FAD alone are sufficient to support magnetosensitivity suggests that *Dm*CRY-CT acts as an adaptor protein, bringing photoactivated FAD close to effectors, possibly HK. Proximity may enable HK to be activated directly by the resultant change in oxidative state that results from the photoactivation of FAD. Very high levels of FAD negate this requirement. In the presence of *Dm*CRY-CT, the amount of photoactivated FAD required is presumably lower and, therefore, more reflective of normal physiological amounts of this flavin.

## The integrity of the CTT is important

**Fig. 3:**
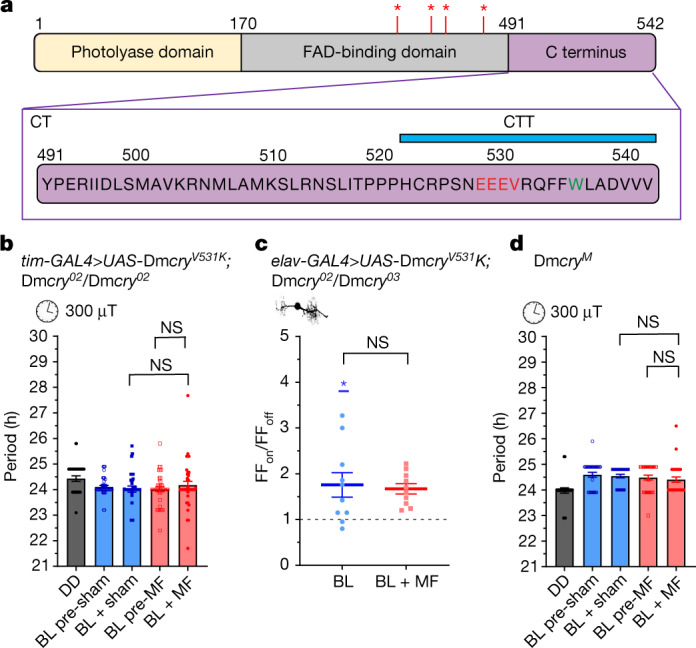
Integrity of the CTT is required for it to facilitate magnetosensitivity. **a**, Schematic of the domain structure of full-length *Dm*CRY, including the CT (amino acids 491–542) and CTT (amino acids 521–542). The four Trp residues, presumed to be essential for the canonical RPM, are indicated by red asterisks. A putative PDZ-binding site (EEEV 528–531, shown in red) was mutated (Val531) *Dm*CRY(V531K). The Trp residue (Trp536) mutated in Luc–CT(W536F) is shown in green. **b**, Dm*cry*^*V531K*^ expressed in clock neurons (*tim-GAL4*) does not support magnetosensitivity in the circadian period-shortening assay. A two-way ANOVA revealed no significant main effects or interaction effects (interaction, *F*_1,52_ = 0.09, *P* = 0.77). *n* = 53 (DD), *n* = 43 (BL pre-sham), *n* = 41 (BL + sham), *n* = 41 (BL pre-MF), *n* = 38 (BL + MF) (Extended Data Table 4a). **c**, Expression of Dm*cry*^*V531K*^ in aCC neurons is sufficient to support BL sensitivity (*t*_9_ = 2.934, *P* = 0.017, *n* = 10; Extended Data Fig. 7a) but not potentiation in the BL + MF condition (100 mT, *t*_18_ = 0.299, *P* = 0.768, *n* = 10). **d**, Expression of Dm*cry*^*M*^, a truncated CRY variant lacking the terminal 19 amino acids, including the PDZ-binding motif (528–531), does not support sensitivity to a 300 µT MF (3 Hz, two-way ANOVA, *F*_1,122_ = 0.021, *P* = 0.89). *n* = 26 (DD), *n* = 31 (BL pre-sham), *n* = 31 (BL + sham), *n* = 32 (BL pre-MF), *n* = 32 (BL + MF) (Extended Data Table 4b). The blue asterisks represent significance values before versus during BL exposure (same cells, two-tailed paired *t*-tests) and the black asterisks represent comparisons of the BL versus BL + MF condition (different cells, unpaired two-tailed *t*-tests). The reported *n* value for each electrophysiological recording is derived from independent cells from biologically independent animals. The reported *n* values for each circadian period derives from biologically independent animals. Data are mean ± s.e.m.

**Extended Data Fig. 7:**
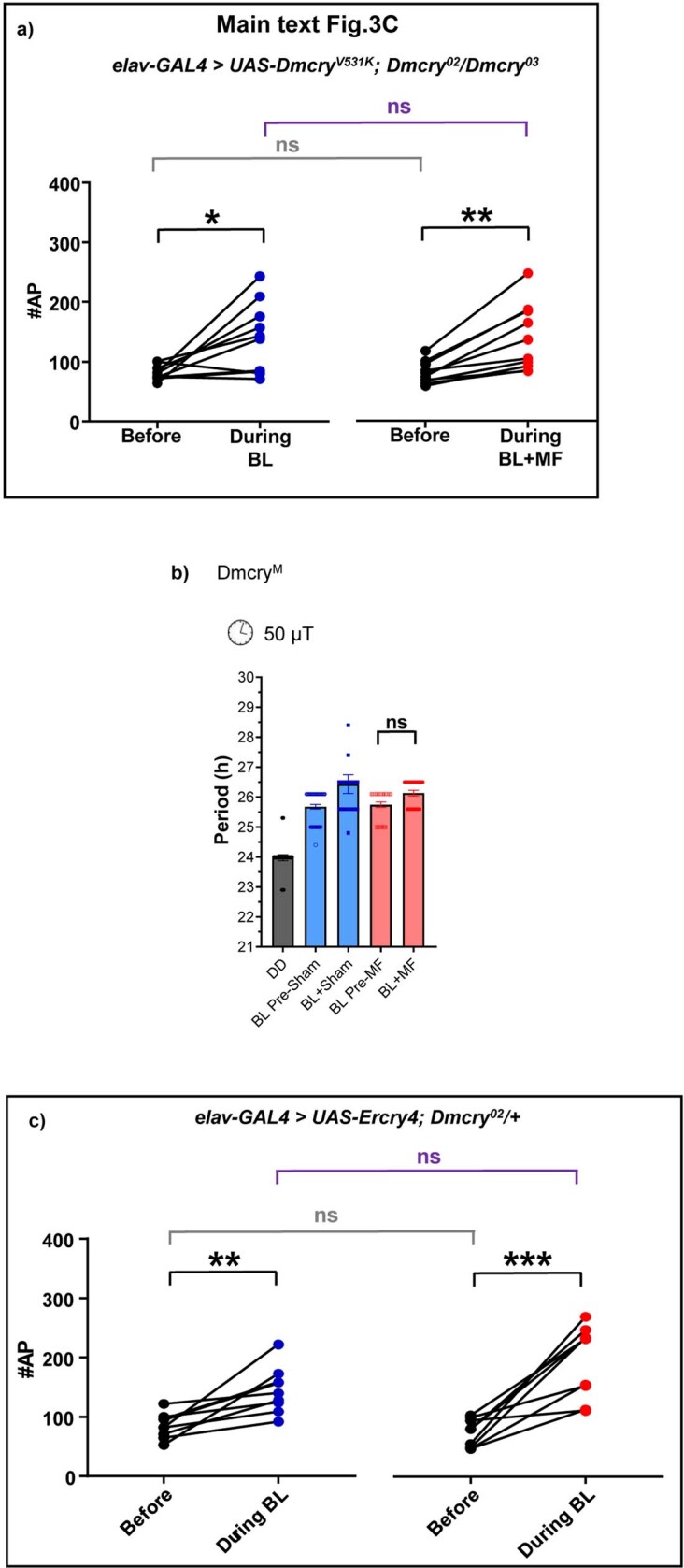
Supporting circadian and electrophysiology data for main text Figs. 3 and 4. (a). Raw AP counts for each aCC neuron recorded expressing *Dmcry*^*V531K*^ in the 15 s before *vs*. the following 15 s during BL±MF, from which the average firing-fold change was derived for data reported in main text Fig. 3c. BL: n = 10, BL+MF: n = 10. Paired t-tests – two tailed, were used to compare before *vs*. during for cells exposed to BL (left hand graph) or to BL±MF exposure (right hand graph). MF-potentiation between the two groups was tested by unpaired t-tests – two tailed (different cells). (b). *Dm*cry^M^ flies do not undergo period shortening following exposure to a 50 µT MF (DD n = 26, BL Pre-Sham n = 51, BL+Sham n = 42, BL Pre-MF n = 53, BL+MF n = 38). (c). Raw AP counts for BL and BL+MF exposure for *Er*CRY4, showing an increase in neuronal excitability to both stimuli (BL: n = 8, BL+MF: n = 9). Paired t-tests – two tailed were used to compare before *vs*. during for cells exposed to BL (left hand graph) or to BL±MF exposure (right hand graph). MF-potentiation between the two groups was tested by unpaired t-tests – two tailed (different cells). The reported n for each electrophysiological recording is derived from independent cells from biologically independent animals. The reported n for each circadian period derives from biologically independent animals. Error bars denote ±SEM, ns p ≥ 0.06, *p ≤ 0.05, **p ≤ 0.01, ***p ≤ 0.001.

The less-robust response from Luc–CT(W536F) (Fig. 1d) suggests that the integrity of the CTT might be important to its role in facilitating magnetosensitivity. The CTT of *Dm*CRY also contains several linear motifs, including putative PDZ-binding sequences^42^ (for example, EEEV 528–531; Fig. 3a). PDZ proteins function as modular scaffolds that direct the cellular localization of signalling molecules, such as ion channels (for example, Shaker K^+^)^43,44^, and the assembly of signalling partners (including *Dm*CRY) into a ‘signalplex’ of the phototransduction cascade in the *Drosophila* eye^21,22^. To examine the importance of the CTT structure and, specifically, to determine whether the putative PDZ-binding motif at residues 528–531 regulates magnetosensitivity, we mutated valine to lysine at position 531 (V531K)^42^ in full-length *Dm*CRY. Pan-circadian expression (that is, using the *tim-GAL4* driver) of Dm*cry*^*V531K*^ in a Dm*cry*^*02*^-null background retained circadian light sensitivity with slight period shortening (24.51 h (DD) versus 24.20 h (BL), *P* = 0.005, Grubbs outlier test excluded a single very weakly rhythmic short period (20.3 h) fly in DD; Fig. 3b and Methods), but did not support a whole-organism behavioural response to MF (interaction, *F*_1,158_ = 0.55, *P* = 0.33; Fig. 3b and Extended Data Table 4a). Expression of Dm*cry*^*V531K*^ in aCC neurons showed the expected effect of BL on action potential firing (1.76-fold; Fig. 3c and Extended Data Fig. 7a). As in the circadian assay, this variant was unable to support magnetosensitivity in the aCC neuron (100 mT, *P* = 0.77; Fig. 3c). The loss of an MF effect, but the retention of a BL response for *Dm*CRY(V531K), is reminiscent of the MF phenotype of the UAS-*Dm*CRY∆ variant^3,4^, which carries a deletion of the CTT^16^. To further validate this result, we used Dm*cry*^*M*^—a variant lacking the final 19 amino acids of the CTT^45^, including the putative PDZ domain centred around residue 531. Expression of Dm*cry*^*M*^ retained circadian light sensitivity but did not show MF-induced period shortening as tested at both 300 µT (3 Hz, *F*_1,122_ = 0.021, *P* = 0.89; Fig. 3d) and 50 µT (3 Hz, *F*_1,180_ = 0.3, *P* = 0.6; Extended Data Fig. 7b and Extended Data Table 4b–d). These results confirm the CTT as a probable mediator of the MF response, where it may serve to facilitate the formation of protein complexes that transduce the magnetic signal.

## *Er*CRY4 supports magnetosensitivity

**Fig. 4:**
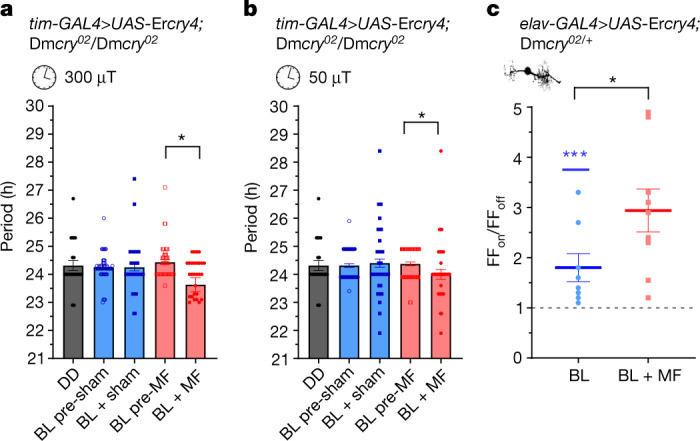
*Er*CRY4 is sufficient to support MF sensitivity in *Drosophila*. **a**, Expression of Er*cry4* in *Drosophila* clock neurons (through *tim-GAL4*) results in significant period shortening in the presence of a 300 µT MF (3 Hz, two-way ANOVA interaction, *F*_1,238_ = 4.4, *P* = 0.036). *n* = 22 (DD), *n* = 49 (BL pre-sham), *n* = 41 (BL + sham), *n* = 49 (BL pre-MF), *n* = 30 (BL + MF) (Extended Data Table 5a–c). **b**, Period shortening is also present in a 50 µT MF (3 Hz, two-way ANOVA interaction, *F*_1,237_ = 3.97, *P* = 0.047). *n* = 22 (DD), *n* = 64 (BL pre-sham), *n* = 59 (BL + sham), *n* = 64 (BL pre-MF), *n* = 54 (BL + MF) (Extended Data Table 5d,e). **c**, Relative firing-frequency recordings of aCC motorneurons expressing Er*cry4* for the BL versus BL + MF condition. BL exposure increases action potential firing (1.8-fold, *t*_7_ = 3.6, *P* = 0.0088, *n* = 8; Extended Data Fig. 7c), an effect that is potentiated by the co-presence of an MF (100 mT, 2.94-fold, BL versus BL + MF, *t*_15_ = 2.17, *P* = 0.046, *n* = 9). The blue asterisks represent significance values for before versus during BL exposure (same cells, paired two-tailed *t*-tests) and the black asterisks represent comparisons of BL versus BL + MF (different cells, unpaired two-tailed *t*-tests). The reported *n* value for each electrophysiological recording is derived from independent cells from biologically independent animals. The reported *n* value for each circadian period derives from biologically independent animals. Data are mean ± s.e.m.

Recent in vitro spectroscopy studies have suggested that CRY4, encoded in the genome of the European robin, *Erithacus rubecula*, a migratory songbird^46^, may represent the magnetoreceptor that is responsible for long-distance navigation in this species. We generated a *UAS-*Er*cry4* transgene and expressed it in clock neurons of the fly. We observed significant period-shortening on exposure to an MF compared with sham treatment at 300 µT MF (3 Hz, two-way ANOVA *F*_1,238_ = 4.4, *P* = 0.036; Fig. 4a) or 50 µT (3 Hz, two-way ANOVA, interaction, *F*_1,237_ = 3.97, *P* = 0.047; Fig. 4b and Extended Data Table 5). Expression of *E. rubecula cry4* (Er*cry4*) in aCC neurons was also sufficient to render the cell sensitive to BL (1.8-fold) and substantially sensitive to an external MF (100 mT, 2.94-fold, *P* = 0.046; Fig. 4c and Extended Data Fig. 7c).

## Conclusions

We have observed that, contrary to several reports^2,14^, but not others^3^, full-length *Dm*CRY is sufficient, but not strictly necessary, to mediate magnetosensitivity. The expression of the C-terminal 52 residues of *Dm*CRY is sufficient to support magnetosensitivity in both single-neuron and whole-animal assays. Our results challenge the canonical CRY-dependent RPM model of animal magnetoreception (based on the requirement for full-length CRY, including FAD binding and the Trp chain), yet are consistent with an RPM. Although it remains unclear whether Luc–CT binds to FAD directly, the Luc–CT response is potentiated by increasing the cytosolic availability of FAD—a common biological redox cofactor—implying that redox reactions are at the core of magnetosensitivity^47^. We cannot exclude that alternative RPs that are not directly photochemically generated may also contribute to magnetoreception, which would be consistent with a growing list of examples reporting RP-mediated magnetoreception in the dark^30,48–50^. The synergistic interaction between Luc–CT and free FAD argues that the former facilitates the formation of a complex that enables the transduction of a magnetically derived signal by the latter. Moreover, free FAD itself can mediate a magnetic response in vivo but at high, non-physiological, levels. We interpret these results to suggest that evolution has shaped the defining element of CRY, its CT, to bring the RP to the proximity of cellular effectors such as HK. Thus, through protein–protein interactions, CRY may potentiate the weak activity of the geoMF on any associated RP. In this regard, the primary role of CRY would be that of a magnetotransducer rather than a magnetoreceptor.

The unexpected observation that robin *Er*CRY4 can also mediate MF effects in *Drosophila*, in both circadian and electrophysiological assays, argues that the fly is an excellent tractable model system to dissect the molecular component of magnetoreception. This has raised the question of why flies have a magnetic sense, given that they do not navigate and migrate in the same way as birds do. Although our circadian phenotype is somewhat contrived and seeks to use a sensitized CRY background (dim constant BL) to provide the best opportunity for observing any MF effects, *Dm*CRY is not only a circadian photoreceptor—it also mediates geotaxis^51^. Independent studies have revealed that geotaxis shows a *Dm*CRY-dependent magnetosensitivity^52,53^. Notable results have suggested that flies exposed to an MF as embryos are imprinted on the MF in which they develop and, as adults, they prefer to forage with downward movement in their home MF^54^. As *D. melanogaster* is well known to forage/mate/oviposit on rotten fruits that are usually found at the ground level, this geotactic magnetic sense would appear to have fitness value.

In conclusion, our observations suggest an ancient and ubiquitous effect of MFs on biological RPs. Through CRY, evolution has optimized such an effect by bringing together two functions, receptor and transductor, that are required for magnetosensing but not necessarily as parts of the same molecule. The fact that *Drosophila* (and other non-migrating animals) can sense external magnetic fields has been reported by many independent groups^55^. This appears reflective of the physiochemical properties of flavins such as FAD to form RPs. In animals that navigate, this mechanism has presumably been adapted to underpin this behaviour, but the underlying physiochemical properties of CRY-dependent magnetosensitivity appear to be shared across navigating and non-navigating animals.

## Methods

### Fly stocks

For larval aCC neuron recordings, embryos were raised at 25 °C under a 12 h–12 h light–dark cycle until third instar wall-climbing larvae (L3) emerged, these were then kept in the dark through the day of recording to minimize light-dependent *Dm*CRY degradation. Recordings were conducted between circadian time hours 2–10. Flies were maintained on standard corn meal medium at 25 °C. The driver line *elav*^*C155*^*-GAL4;* Dm*cry*^*03*^ was obtained by crossing the driver line from Bloomington Stock centre (BL458) into a Dm*cry*^*03*^ background as described previously^56^. *Dmcry*^*02*^ flies were obtained from Bloomington Stock centre (BL86267). *tim-GAL4;cry*^*02*^ and *UAS-*Dm*cry;*Dm*cry*^*02*^ were described previously^3,57^. Dm*cry*^*M*^ (supplied by D. Dolezel) has a stop codon inserted at amino acid 523 and lacks the final 19 amino acids of the CTT, which includes the putative PDZ-binding motif at 531^45^ (Fig. 3a). The generation of new transgenic flies for this study is described below.

### Molecular cloning of Luc–CT

The luciferase coding sequence was cloned from the *UAS-Luc-*Dm*cry* fly line^18^ and subsequently amplified using the following primers to include overhangs compatible for the NEB Gibson Assembly assay: F, TATCCTTTACTTCAGGCGGCCGCATGGAAGACGCCAAAAACATAAAGAAAGG; R, TCCGGATACTCGAGCACGGCGATCTTTCCGCCC.

The CT portion of Dm*cry* was produced by gene synthesis (GeneArt, Thermo Fisher Scientific) based on the original *GFP-CT* construct^3^. *CT* was designed to include 5′ and 3′ overhangs compatible with subsequent NEB Gibson Assembly assay (sequence, AAAGATCGCCGTGCTCGAGTATCCGGAGCGGATCATTGATTTGTCCATGGCCGTGAAGCGCAACATGCTGGCCATGAAGTCCCTGCGCAACAGCCTGATCACCCCCCCACCACATTGCCGCCCCAGCAATGAGGAGGAAGTGCGCCAGTTCTTCTGGCTGGCCGATGTGGTGGTGTAATCTAGAGGATCTTTGTGAAGGA).

The pJFRC2-10XUAS-IVS-mCD8::GFP (Addgene, 26214) plasmid was digested with NotI and XbaI. A Gibson Assembly assay was performed to ligate pJFRC, *Luc* and *CT* in a single-step reaction. A Myc tag was produced by annealing single oligos designed using the Oligator software (https://gcat.davidson.edu/iGem10/) and ligated 5′ of Luc-CT. In brief, 5 µM of the oligos 47-mer Top1, 5′-GATCTCACAATGGAACAGAAGCTGATCTCCGAGGAGGACCTGGGCGC; 47-mer Bottom1, 5′ GGCCGCGCCCAGGTCCTCCTCGGAGATCAGCTTCTGTTCCATTGTGA, resulting in 5′ BglII and 3′ Notl overhangs once annealed, were diluted in 1× annealing buffer (0.1 M NaCl; 10 mM Tris-HCl, pH 7.4), boiled in 500 μl of H_2_O for 10 min and left overnight to cool down to room temperature to hybridize. The pJFRC2-10XUAS-Luc-CT plasmid was then digested with BglII and NotI. The fragment encoding the Myc tag was then ligated using standard methods. After sequence validation, the plasmid was injected into the *y* *w,* *M(eGFP,vas-int,dmRFP)ZH-2A;P{CaryP}attP40* (stock 13–40, Cambridge University Fly facility) using the Phi31 integrase system for insertion. The resulting transformants were subsequently backcrossed into the *w*^*1118*^ background for seven generations. The Luc–CT(W536F) transgene was generated by gene synthesis (Eurofins) and subcloned into pJFRC-MUH through the 5′ NotI and 3′ XbaI restriction sites. Transgenic injections for Luc–CT(W536F) were carried out by Manchester University Fly Facility using the same *y* *w* *M(eGFP,vas-int,dmRFP)ZH-2A;P{CaryP}attP40* line (stock 13–40, University of Cambridge Fly Facility). The Er*cry4* transgenic was generated by gene synthesis (NBS Biological) and subcloned into pJFRC-MUH through the NotI and KpnI restriction sites. Injection was carried out by Bestgene into *y* *w;PBac{y[+]-attP-3B}VK00002* (Bloomington, 9723) using the Phi31 integrase system.

*HA-*Dm*cry*^*V531K*^ (containing a HA tag at the N terminus of the encoded protein) was already available as a clone in the yeast plasmid pEG202 (ref. ^42^). It was released by EcoRI-XhoI digestion and subcloned into pUAST^58^. Transgenics were produced by P-element transformation by the University of Cambridge Fly Facility using the line S(6)1 inserted on chromosome 2.

### Electrophysiology

The experimenter was blinded to genotype during both recordings and subsequent data analysis. L3 larvae were dissected under extracellular saline as described^59^ with the only modification being a red filter applied to both the dissecting light and compound microscope to minimize *Dm*CRY degradation before and during recordings. Thick-walled borosilicate glass electrodes (GC100F-10; Harvard Apparatus) were fire-polished to resistances of 10–15 MΩ. Recordings were made using the Multiclamp 700B amplifier controlled by pCLAMP (v.10.4) and the Digidata 1440A analogue-to-digital converter (Molecular Devices). Only cells with an input resistance of ≥500 MΩ were used. Traces were filtered at 10 kHz and sampled at 20 kHz. The extracellular saline solution contained the following: 135 mM NaCl, 5 mM KCl, 4 mM MgCl_2_.6H_2_O, 2 mM CaCl_2_.2H_2_O, 5 mM TES and 36 mM sucrose, pH 7.15. The intracellular patch solution contained the following (in mM): 140 mM potassium-d-gluconate, 2 mM MgCl_2_.6H_2_O, 2 mM EGTA, 5 mM KCl and 20 mM HEPES, pH 7.4. KCl and CaCl_2_ were from Thermo Fisher Scientific; sucrose was from BDH; all of the remaining chemicals were from Sigma-Aldrich. Mecamylamine (1 mM) was applied to all preparations to isolate the aCC motoneurons from excitatory cholinergic synaptic input. For recordings supplemented with additional FAD or riboflavin (Sigma-Aldrich), dilutions were made up in intracellular saline and kept in the dark.

### Photoactivation and magnetic field application

Light stimulation was supplied by a blue LED (470 nm, Cairn Research) at a power of around 2.2 mW cm^−2^, a value used previously to stimulate *Dm*CRY^60^. Each cell was injected with a variable amount of constant current until the threshold potential was reached and the neuron was allowed to settle, for some minutes, until action potential firing was stable at ~5–7 Hz. Once a stable firing rate was achieved, each neuron was recorded for at least 20 s before exposure to BL illumination for 30 s. No significant change to the action potential firing rate was observed without BL illumination. Magnetic exposure was provided by two NdFeB static magnets mounted around the preparation at a distance that provided an MF of 100 mT (±5 mT). Field strength was measured using the 5180 Gauss/Tesla Meter (F.W. Bell). This method is essentially identical to that used previously^4^.

### Statistical analysis of electrophysiological recordings

The D’Agostino–Pearson analysis showed our data to be normally distributed and parametric tests were therefore applied in all cases. Data are shown as mean ± s.e.m. To determine BL sensitivity, paired two-tailed *t*-tests were used to compare the number of action potentials that a neuron fired in the 15 s after light stimulation versus the number of action potentials in the 15 s before light exposure. For a comparison between BL and BL + MF, the number of action potentials in the 15 s before and after BL or BL + MF exposure was used to determine the firing fold change (FF_on_/FF_off_) for each cell. Statistical significance for MF potentiation against the BL effect alone was determined using unpaired two-tailed *t*-tests to compare the firing fold change for the BL dataset versus the BL + MF dataset. In cases in which multiple genotypes/conditions were tested simultaneously, two-way ANOVA with Newman–Keuls post hoc testing was used. For the FAD dose–response curve, a BL effect of [FAD] was determined by linear regression fitting and significance was determined using an ANCOVA model. Average MF potentiation of the Luc–CT FAD dose–response (Fig. 2a) was determined on the basis of the intercept of the *y* axis. Unpaired two-tailed *t*-tests were also applied in the Extended Data (Figs. 1a–c, 3b,c, 4a–d, 5a–c and 7a,c) to compare the number of action potentials in the before BL ± MF conditions, as well as to BL and BL + MF exposures. Control lines were also compared to their respective experimental genotype by both one-way ANOVA (with BL and BL + MF recordings separated) and by two-way ANOVA. Raw data are reported in the Extended Data (Figs. 1a–c, 3b,c, 4a–d and 7a,c).

### Behavioural analyses and statistics

Circadian locomotor activity was recorded using a *Drosophila* Trikinetics Monitor 2 (ref. ^3^). To test the effects of MF on the free-running circadian period of locomotor activity, we used a modified version of the Schuderer apparatus, which consists of two independent double-wrapped coils placed inside two mu-metal boxes within a commercial incubator. The shielded four quadratic Helmholtz coil systems produce a homogenous, linearly polarized, *B* field with perpendicular orientation to the horizontal plane of the Trikinetics monitors. Each coil is formed with a pair of wires, with the current passing in the same direction through both wires for MF exposure but in opposite directions to provide a sham exposure condition (0 T). A computer randomly assigns the MF- and sham-exposed chambers and the experiment is performed in a blinded manner^17^.

Flies (aged 1–3 days) were first entrained at 20 °C in the apparatus under a dim BL–DD 12 h–12 h cycle for 3 days, before being pre-exposed to continuous BL for 7 days, followed by exposure to BL + MF or BL + sham for a further 7 days^3^. Thus, there were four measurements, the pre-exposure (BL) period of flies that were subjected to an MF or sham, plus the exposure period for both (BL + MF and BL + sham). A fifth control condition examined the period of *Luc-CT;*Dm*cry*^*02*^ flies in DD without exposure. All of the experiments were performed using a low-frequency 3 Hz field at 300 μT or 50 μT and dim BL at 0.15–0.25 μW cm^−2^, wavelength 450 nm, 40 nm broad range (RS Components). The driver *tim-GAL4* was used to express UAS-Dm*cry* transgenes as previously described^3^. Locomotor data were collected in 1 min time bins but analysed in 10 and 30 min bins.

Rhythmicity and period were determined using spectral analysis using a MATLAB-based version of the BeFly program^60^. Statistical analysis of period was performed using ANOVA with either Statistica (Statsoft) for factorial analyses or Prism (GraphPad) for one-way ANOVA. Although there was a clear prediction that Luc–CT flies would have a shorter period under an MF^3^, we nevertheless used the stringent Newman–Keuls post hoc test to compare groups after factorial ANOVA with the more liberal Fisher LSD test. To compare the DD periods with those from the BL pre-exposure conditions, we used unpaired *t*-tests. Circadian data were first tested using a Grubb’s outlier test (GraphPad Prism, alpha = 0.01, two-sided, *Z* = 5.3). One datum from the DD data of *Dm*CRY(V531K) that represented the least-robust single period in the dataset with an anomalous period of 20.3 h (8 s.d. away from the mean) was identified and removed.

### Determination of sample size

Sample size for electrophysiological recordings was based on preliminary experiments as well as published work^4^, which showed reproducible effects. For weaker effects, sample size was increased. For circadian work, sample size was based on previous experience and extensive published work^3^ as well as a power calculation using the s.d. of preliminary experiments. This indicated, with a power of 80% at a 95% confidence, that we could reliably detect a 0.4 h difference in period with an *n* = 16, and a 0.3 h difference with an *n* = 28. All comparisons of sham and electromagnetic field exposure before and after had an *n* > 28.

### Replication

Electrophysiological experiments were repeated twice with the following exceptions: (1) for Fig. 2a, the FAD dose–response curve due the already high number of recordings for the overall experiment (*n* = 62); (2) for Fig. 2c, riboflavin (50 μM) manipulation in the presence of Luc–CT; and (3) for Fig. 2b,d, 50 μM FAD and 200 μM exposure in the Dm*cry*-null background (however, the effect size for 200 μM FAD (Fig. 2d) was increased to *n* = 20) and (4) for *Er*CRY4, in which the effect was clear and consistent as to not require further laborious recordings. Circadian MF experiments were repeated at least twice.

### Reporting summary

Further information on research design is available in the Nature Portfolio Reporting Summary linked to this article.

## Online content

Any methods, additional references, Nature Portfolio reporting summaries, source data, extended data, supplementary information, acknowledgements, peer review information; details of author contributions and competing interests; and statements of data and code availability are available at 10.1038/s41586-023-05735-z.

### Supplementary information

Reporting Summary

## Figures and Tables

**Extended Data Table 1:**
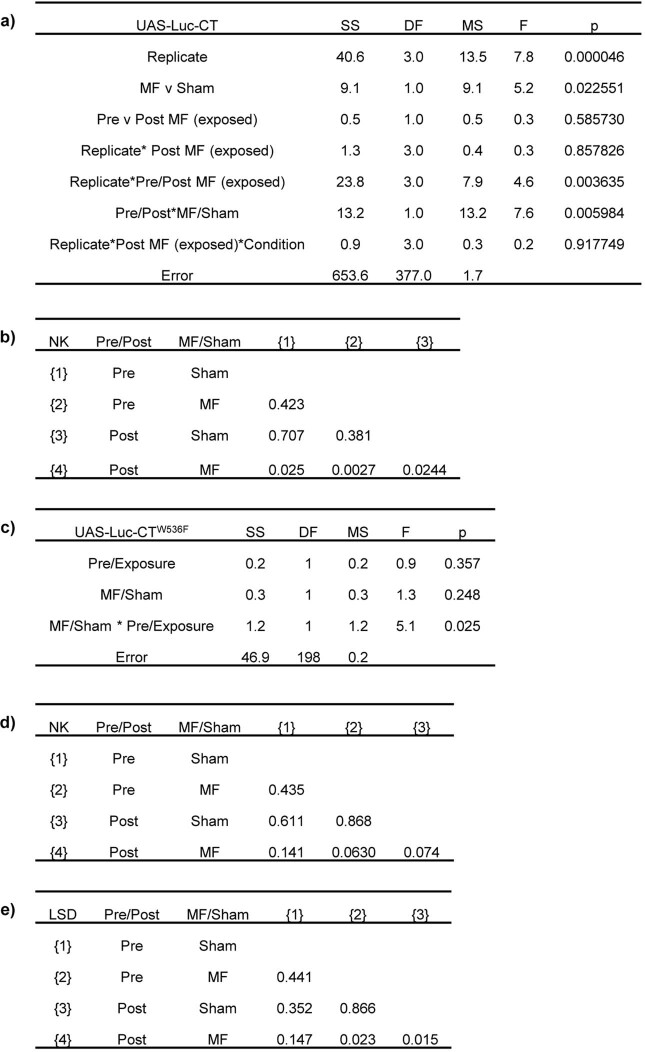
Supporting circadian data for main text Fig. 1

(a). Table of 3-way ANOVA of BL conditions reveals a significant Sham / MF x pre / post-exposure interaction (F_1,377_ = 7.6, p = 0.006) with MF (300 µT) exposed flies expressing Luc-CT, showing a significantly shorter period than sham exposed flies. (b). Table of Newman-Keuls *post-hoc* comparisons from a. (c). Table of a 2-way ANOVA of Luc-CT^W536F^ circadian experiment reveals a significant interaction (F_1,198_ = 5.1, p = 0.025) (d). Tables of Newman-Keuls *post-hoc* and (e). Fisher LSD comparisons reveal a MF field effect on Luc-CT^W536F^ circadian period in the latter test. The relevant comparisons in the more stringent NK test approach significance.

**Extended Data Table 2:**
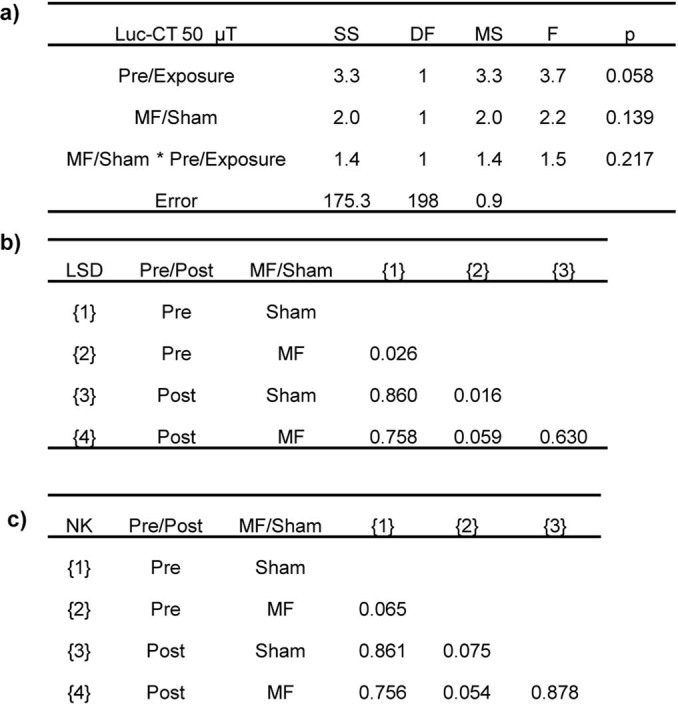
Supporting circadian data for main text Fig. 1

(a). Exposure of Luc-CT to a 50 µT (3 Hz) MF results in a small reduction in period compared to sham (DD n = 28, BL Pre-Sham n = 52, BL+Sham n = 50, BL Pre-MF n = 52, BL+MF n = 48. 2-way ANOVA table (interaction F_1_,_198_ = 3.7, p = 0.056) on circadian period for Luc-CT at 50 µT. (b). LSD and (c). Newman-Keuls *post-hoc* tests reveal a marginal significant effect (p = 0.059, 0.054, respectively) on period shortening.

**Extended Data Table 3:**
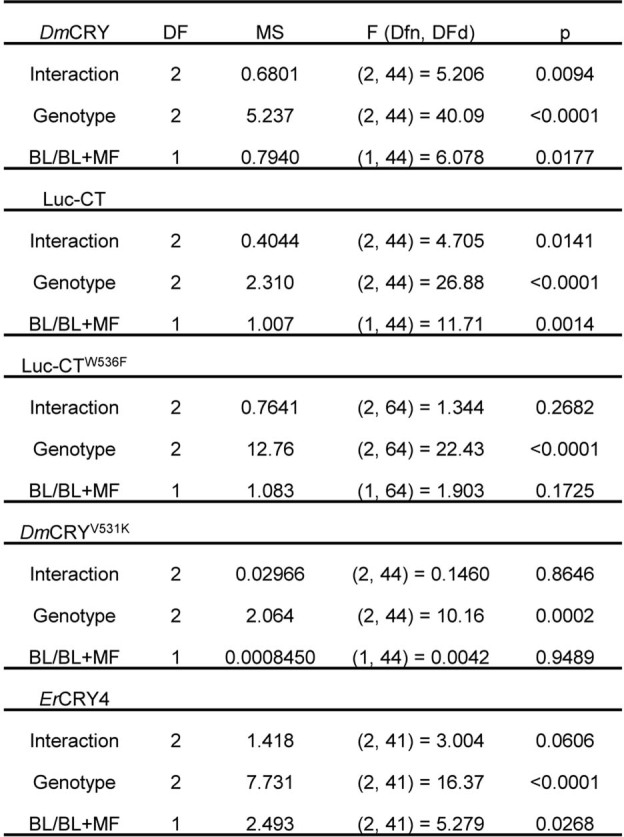
Supporting electrophysiological controls

A 2-way ANOVA comparing FF changes between the parental (i.e. un-driven) *w; UAS-cry-*transgene and *elav-GAL4;* both in *Dmcry*^*02*^*/Dmcry*^*03*^ null background, *vs*. the expressed *cry* transgene (in which both *GAL4* and UAS elements are combined to express the *cry-transgene*). Additionally, a BL *vs*. BL+MF comparison was also performed.

**Extended Data Table 4:**
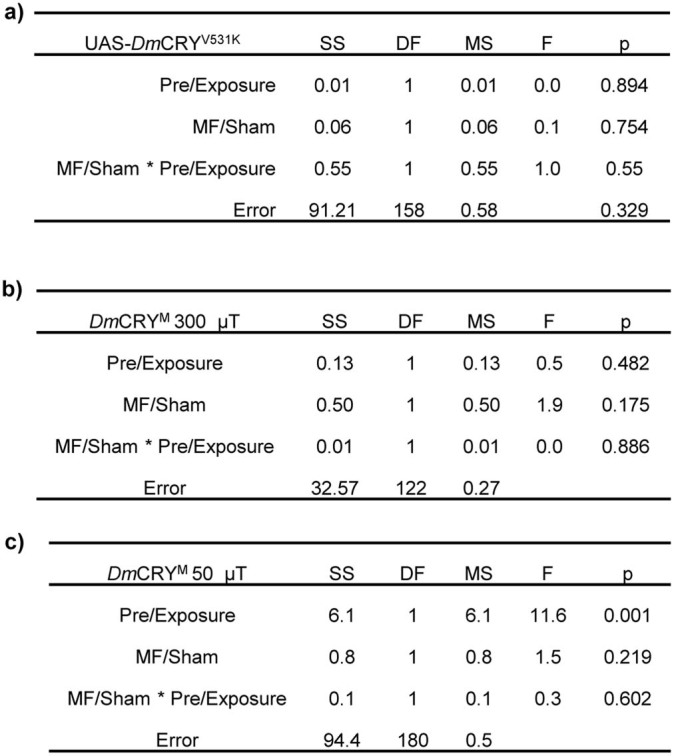
Supporting circadian data for main text Fig. 3

(a). Table of 2-way ANOVA of *Dm*CRY^V531K^ circadian period shortening. No significant main differences nor interaction effects were observed (F_1,158_ = 0.55, p = 0.33). (b). Summary of the 2-way ANOVA of the *Dm*CRY^M^ variant which lacks the PDZ binding motif and fails to support period shortening in a MF at 300 µT (3Hz, interaction F_1,122_ = 0.021, p = 0.89. (c). 2-way ANOVA of *Dm*CRY^M^ flies at 50 µT (3Hz) shows no significant MF based interaction F_1_,_180_ = 0.3, p = 0.6).

**Extended Data Table 5:**
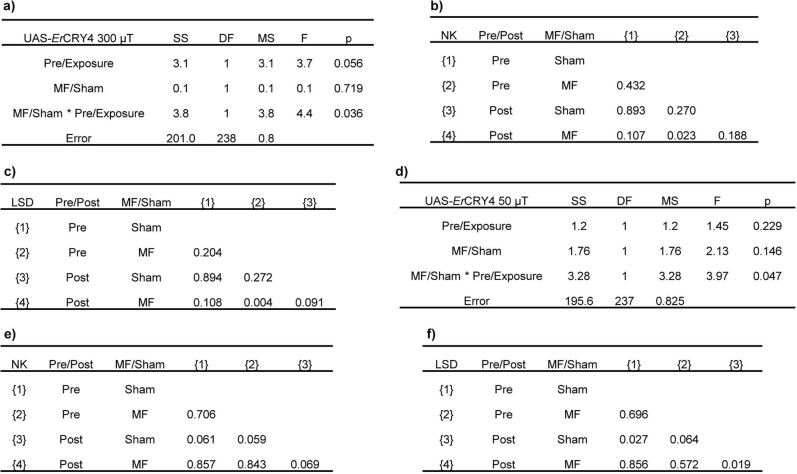
Circadian and data for *Er*CRY4 in main text Fig. 4

(a). 2-way ANOVA reveals a significant Sham/MF x pre/post-exposure interaction and a period shortening following exposure to a 300 µT / 3 Hz MF. (b). A Newman-Keuls and (c). Fisher LSD *post-hoc* tests for *Er*CRY4 at 300 µT / 3 Hz MF. (d). A 2-way ANOVA also reveals a significant MF effect at 50 µT / 3 Hz for *Er*CRY4. (e). Newman-Keuls and (f). Fisher LSD *post-hoc* tests for *Er*CRY4 at 50 µT / 3 Hz MF exposure.

## Data Availability

The analysed data are included in Extended Data Figs. 1–7 and Extended Data Tables 1–5. All raw data for circadian locomotor experiments have been deposited in the Open Science Forum repository (https://osf.io/6fnra/?view_only=1b825d853813402f9aa41927e5c3cc0f). Raw data for the electrophysiology experiments are available on request.
