# Publisher Correction: Essential elements of radical pair magnetosensitivity in *Drosophila*

**DOI:** 10.1038/s41586-023-05929-5

**Published:** 2023-03-15

**Authors:** Adam A. Bradlaugh, Giorgio Fedele, Anna L. Munro, Celia Napier Hansen, John M. Hares, Sanjai Patel, Charalambos P. Kyriacou, Alex R. Jones, Ezio Rosato, Richard A. Baines

**Affiliations:** 1 Division of Neuroscience, School of Biological Sciences, Faculty of Biology, Medicine and Health, University of Manchester, Manchester Academic Health Science Centre, Manchester, UK; 2 Department of Genetics and Genome Biology, University of Leicester, Leicester, UK; 3 Department of Biology, Ecology and Earth Sciences (DiBEST), University of Calabria, Rende, Italy; 4 Pelican Healthcare, Cardiff, UK; 5 Manchester Fly Facility, Faculty of Biology, Medicine and Health, University of Manchester, Manchester, UK; 6 Biometrology, Chemical and Biological Sciences Department, National Physical Laboratory, Teddington, UK

**Keywords:** Molecular neuroscience, Sensory processing

Correction to: *Nature* 10.1038/s41586-023-05735-z Published online 22 February 2023

In the version of this article initially published, the Author contributions section was truncated. The first two sentences have been corrected to read “R.A.B., A.A.B., G.F., A.R.J., C.P.K. and E.R. conceived the project. A.A.B., G.F., A.L.M. and C.N.H. performed experiments” in the HTML and PDF versions of the article.

