# Supplementary material for: Essential elements of radical pair magnetosensitivity in Drosophila
Source: Nature. 2023 Feb 22;615(7950):111–6. doi: 10.1038/s41586-023-05735-z (PMC9977682; doi:10.1038/s41586-023-05735-z)
Supplement: Supplementary file 1 — Reporting Summary [file 41586_2023_5735_MOESM1_ESM.pdf]

nature portfolio

Corresponding author(s): Richard Baines

Last updated by author(s): Jan 5, 2023

Reporting Summary

Nature Portfolio wishes to improve the reproducibility of the work that we publish. This form provides structure for consistency and transparency in reporting. For further information on Nature Portfolio policies, see our Editorial Policies and the Editorial Policy Checklist.

Statistics

For all statistical analyses, confirm that the following items are present in the figure legend, table legend, main text, or Methods section.

n/a	Confirmed	
☐	☒	The exact sample size (n) for each experimental group/condition, given as a discrete number and unit of measurement
☐	☒	A statement on whether measurements were taken from distinct samples or whether the same sample was measured repeatedly
☐	☒	The statistical test(s) used AND whether they are one- or two-sided<br>*Only common tests should be described solely by name; describe more complex techniques in the Methods section.*
☒	☐	A description of all covariates tested
☐	☒	A description of any assumptions or corrections, such as tests of normality and adjustment for multiple comparisons
☐	☒	A full description of the statistical parameters including central tendency (e.g. means) or other basic estimates (e.g. regression coefficient) AND variation (e.g. standard deviation) or associated estimates of uncertainty (e.g. confidence intervals)
☐	☒	For null hypothesis testing, the test statistic (e.g. F, t, r) with confidence intervals, effect sizes, degrees of freedom and P value noted<br>*Give P values as exact values whenever suitable.*
☒	☐	For Bayesian analysis, information on the choice of priors and Markov chain Monte Carlo settings
☒	☐	For hierarchical and complex designs, identification of the appropriate level for tests and full reporting of outcomes
☒	☐	Estimates of effect sizes (e.g. Cohen's d, Pearson's r), indicating how they were calculated

Our web collection on statistics for biologists contains articles on many of the points above.

Software and code

Policy information about availability of computer code

Data collection	Clampex v10 (Molecular Devices); DAM system 3 Acquisition (Trikinetics), Control system for Schuderer equipment (IT'IS foundation, Switzerland).
Data analysis	Clampfit v10 (Molecular Devices); GraphPad v9 (Prism), DAM file scan (Trikinetics), Statistica v13 (Statsoft) Excel 2016 (Microsoft), BeFly (Delfino L, Campesan S, Fedele G, Green EW, Giorgini F, Kyriacou CP, et al. Visualization of Mutant Aggregates from Clock Neurons by Agarose Gel Electrophoresis (AGERA) in Drosophila melanogaster. Methods Mol Biol 2022;2482:373-383), implemented in MATLAB v9(MathWorks).

For manuscripts utilizing custom algorithms or software that are central to the research but not yet described in published literature, software must be made available to editors and reviewers. We strongly encourage code deposition in a community repository (e.g. GitHub). See the Nature Portfolio guidelines for submitting code & software for further information.

Data

Policy information about availability of data

All manuscripts must include a data availability statement. This statement should provide the following information, where applicable:

- Accession codes, unique identifiers, or web links for publicly available datasets
- A description of any restrictions on data availability
- For clinical datasets or third party data, please ensure that the statement adheres to our policy

All analysed data is included in the extended data section. Raw data pertaining to period shortening has been deposited at: https://osf.io/6fnra/?view_only=1b825d853813402f9aa41927e5c3cc0f. Raw data pertaining to electrophysiology will be made available on reasonable request.

Field-specific reporting

Please select the one below that is the best fit for your research. If you are not sure, read the appropriate sections before making your selection.

☒ Life sciences ☐ Behavioural & social sciences ☐ Ecological, evolutionary & environmental sciences

For a reference copy of the document with all sections, see nature.com/documents/nr-reporting-summary-flat.pdf

Life sciences study design

All studies must disclose on these points even when the disclosure is negative.

Sample size	Sample size for electrophysiological recordings was based on preliminary experiments as well as published work (e.g. Giachello et al 2016, Ref: 4 of the manuscript), which showed reproducible effects. For weaker effects, sample size was increased. For circadian work sample size was based on previous experience and extensive published work (Fedele et al, 2014 PLoS Genetics, Ref: 3 of the manuscript) as well as a power calculation using the SD of preliminary experiments. This indicated, with a power of 80% at a 95% confidence, we could reliably detect a 0.4 hour difference in period with an n=16, and a 0.3 hour difference with an n=28. All comparisons of Sham/EMF/Pre/Post had an n > 28.
Data exclusions	Poor quality electrophysiological recordings were excluded prior to the investigator being unblinded. Such exclusions were based on previously established criteria such as input resistance (e.g. less than 500MOhm: indicative of a poor seal between the electrode and the cell membrane), as described in the methods. For behavioural analyses dead flies were excluded, as well as a single fly based on its highly abnormal period length and weak rhythmicity, this was highlighted via a Grubbs outlier test (and is described in manuscript).
Replication	Electrophysiological experiments were repeated twice with the following exceptions i) Fig2A: FAD dose response curve due the already high number of recordings for the overall experiment (n=62); ii) Fig 2C Riboflavin (50 uM) manipulation in the presence of CRY CT and iii) Fig 2B, D: 50 uM FAD and 200 uM exposure in the Cry null. However, the effect size for 200 uM FAD (Fig 2D) was increased to n=20. iiii) ErCry4 in which the effect was clear and consistent as to not require further recordings. Circadian experiments were repeated at twice. Replication was successful.
Randomization	For behavioural experiments a computer randomly assigns the MF and Sham exposed chambers and the experiment is performed blinded from the investigator, as described previously in Fedele et al 2014 (Ref: 3 of the manuscript) and in the methods.
Blinding	The investigator(s) was blinded to genotype and/or flavin supplement until data collection and analysis were fully completed for each experiment reported.

Reporting for specific materials, systems and methods

We require information from authors about some types of materials, experimental systems and methods used in many studies. Here, indicate whether each material, system or method listed is relevant to your study. If you are not sure if a list item applies to your research, read the appropriate section before selecting a response.

Materials & experimental systems

n/a	Involved in the study	
☒	☐	Antibodies
☒	☐	Eukaryotic cell lines
☒	☐	Palaeontology and archaeology
☐	☒	Animals and other organisms
☒	☐	Human research participants
☒	☐	Clinical data
☒	☐	Dual use research of concern

Methods

n/a	Involved in the study	
☒	☐	ChIP-seq
☒	☐	Flow cytometry
☒	☐	MRI-based neuroimaging

Animals and other organisms

Policy information about studies involving animals; ARRIVE guidelines recommended for reporting animal research

Laboratory animals	Drosophila melanogaster strains used:<br>elavC155-GAL4; ; cry03<br>; tim-GAL4; cry02<br>UAS-DmCry<br>UAS-DmCryV(531)K<br>UAS-Luc-CT<br>UAS-Luc-CT W(536)F<br>UAS-ErCry4<br>w-; ; cry02<br>w-: : cry03<br>; ; cryM

	Electrophysiology: wall-climbing third instar larvae of both sexes used.<br>Circadian period: adult males (1-3 days old)
Wild animals	No wild animals were used in the study
Field-collected samples	No field collected samples were used in the study.
Ethics oversight	Study did not require an ethical approval.

Note that full information on the approval of the study protocol must also be provided in the manuscript.
